# Out-of-pocket health expenditures in patients living with ınborn errors of metabolism

**DOI:** 10.1186/s13023-023-02775-6

**Published:** 2023-07-06

**Authors:** Mehmet Gündüz, Yasemin Yüksel Güdek, Çiğdem Seher Kasapkara

**Affiliations:** 1 Department of Pediatric Metabolism, Ankara Bilkent City Hospital, University of Health Sciences, Ankara, Turkey; 2 Department of Pediatrics, Ankara Bilkent City Hospital, University of Health Sciences, Ankara, Turkey; 3 Department of Pediatric Metabolism, Ankara Bilkent City Hospital, Ankara Yıldırım Beyazıt University, Ankara, Turkey

## Abstract

**Aim:**

The implementation of newborn screening programs for inborn errors of metabolism has advanced the diagnosis and management of affected infants and undoubtedly improved their outcomes. We aimed to determine out-of-pocket health expenditures of patients with inborn errors of metabolism during follow-up and treatment processes and to determine the economic burden on the families.

**Materials and methods:**

A total of 232 patients who voluntarily agreed to participate in the study and were regularly followed up in the Department of Pediatric Metabolism with the diagnosis of Inborn Errors of Metabolism between April 2022 and July 2022 were included. Questionnaires were asked about the demographic characteristics of patients, use of health services, follow-up, treatment procedures, frequency of controls and health expenditures.

**Results:**

The average out-of-pocket expenditure of the households in the last month was 1039.22 ± 1030.08 (minimum: 20, maximum: 5000) Turkish Liras. When we consider the catastrophic health expenditure rate as expenditure exceeding 40% of household income, we found that 9.9% (23 people) of parents included in the study made catastrophic health expenditures. The rate of catastrophic expenditure of patients with a diagnosis of Amino Acid Metabolism Disorders was found to be higher than that of patients with a diagnosis of Vitamin and Cofactor Metabolism Disorders. Similarly, patients with a diagnosis of lysosomal storage diseases had more expenditures than patients with a diagnosis of vitamin and cofactor metabolism disorders. When we compared the rate of catastrophic health expenditure of the patients with urea cycle disorders and the patients with a diagnosis of vitamin and cofactor metabolism disorders, the former had more expenditure than the latter (*p* < 0.05). There was no significant difference between other disease groups in terms of catastrophic expenditure. The rate of catastrophic expenditures of the households living as large family type were higher than the families living as nuclear family type (*p* < 0.01). A statistically significant difference was found between the rates of catastrophic expenditures of the families living in Ankara and those who were admitted from other provinces for follow-up and treatment (*p* < 0.001). However, there was no difference between the rates of catastrophic expenditure of the patients who received any treatment and those who were followed up without treatment (*p* > 0.05).

**Conclusion:**

Due to the high rate of consanguineous marriages in our country, the development of newborn screening programs, the increase in awareness about metabolic diseases and the improvement in diagnostic methods, the frequency of metabolic diseases is increasing, and mortality and morbidity rates are significantly reduced with early diagnosis and treatment opportunities. It is necessary to carry out more comprehensive studies to determine and prevent the socioeconomic effects of out-of-pocket health expenditures of patients living with Inborn Errors of Metabolism.

## Introduction

Inborn errors of metabolism, characterized by disruption of metabolic pathways due to deficient enzymes, cofactors, or transporters, also known as inherited metabolic diseases, constitute a group of diseases presenting with different signs. They are individually rare but collectively common. Newborn screening programs provide the opportunity for early treatment, leading to reduced morbidity and mortality. National newborn screening program for phenylketonuria and biotinidase deficiency was conducted by Ministry of Health with a screening rate more than 97%. Two laboratories of Refik Saydam National Public Health Institution of Turkey (PHIT) situated in Ankara and İstanbul have been used for this purpose. In near future Ministry of Health planned to do expanded newborn screening by MS/MS, making it possible to screen for 40–50 conditions using a single blood spot and by this way the diseases will have greater chance to be determined. Phenylketonuria (PKU) and biotinidase deficiency (BD) are metabolic disorders that are commonly encountered in Turkey. Newborn screening (NBS) for PKU was implemented in our country on 1986 by the collaboration of Ministry of Health and Universities. The process of conducting National Newborn Screening for PKU by one center, the Ministry of Health on December 2006 and BD were added to the program on October 2008 [1].

Early diagnosis and prompt intervention enable patients to avoid the progression of mental disability. Restrictive dietary treatment remains the main therapy, although new emerging treatment options allow patients to be diet free in certain diseases. Families of children diagnosed with IEM experience multifaceted stresses, not only the emotional stress of their child facing a potentially life-threatening diagnosis but also the financial burden the family will experience related to the child’s diagnosis and treatment. In Turkey, health insurance programs and national reimbursement systems are mostly available to support the treatment of children with inborn errors of metabolism (IEM). Prior to the health system reforms, Turkey had a fragmented health insurance system that consisted of five different health insurance schemes, each with a different benefits package: Social Insurance Organization for formal sector employees, Government Employees Retirement Fund for retired civil servants, Active Civil Servants Insurance Fund for civil servants in work and their dependents, Bağ-Kur for artisans, self-employed and agricultural workers and Green Card insurance scheme for the poor. One of the most important variables is poverty in this study. Green card users, according to Turkish law, are those “who are residing in Turkey, who are not covered by any social security institution and do not work in an income-generating job.” Therefore, green card holders are given to people who are not under any social security and are unable to cover their health expenses [2].

They have been set up to specifically cover most of the expenditures by providing dietary formulas and cash reimbursement. Other expenditures, such as specific medications, transportation and health care visits called out-of-pocket costs (OOPCs), must be covered by the patients themselves [3, 4]. Cost of illness studies especially analyse direct costs (goods, services and other resources that are consumed during the provision of health intervention) and indirect costs (production losses due to abseenteeism of the patient or caregiver). The current study aimed to evaluate the economic burden of IEM in Turkey from the payer perspective based on the out-of-pocket expenses of parents and caregivers.

## Methods

This cross-sectional noninterventional study was conducted between April 2022 and July 2022 in Ankara Bilkent City Hospital. The hospital is based in Ankara province, which is the capital city of Turkey. A total of 232 patients with inherited metabolic diseases during their regular outpatient examination with IEM attented to the Department of Pediatric Metabolism were enrolled in this study. Patients who were not under regular follow-up, aged over 18 years and had missing data were excluded from the study. All procedures were in accordance with the ethical standards of the local ethical committee of Ankara Bilkent City Hospital (30/03/2022-E2-22-1428).

The medical and nonmedical financial burdens were evaluated by using a questionnaire to determine the out-of-pocket costs of parents and caregivers of parents with IEM. We accepted the World Health Organization definition of catastrophic health expenditures (CHE), which is out-of-pocket payments for health care that exceed 10% of household expenditures or 40% of subsistence expenditures [4].

Signed informed consent was obtained prior to any interview. The parents or caregivers were invited for a face-to-face interview after routine follow-up examination. Each interview was conducted by the same researcher. We collected demographic features, medical history, financial status of the family and all the economic costs from the parents or caregivers.

### Statistical analysis

All data were processed with IBM SPSS Statistics Version 22. Descriptive analyses were performed, and data are presented as the median (interquartile range [IQR]) and mean (SD) for nonnormally and normally distributed data, respectively. Values were considered significant if *p* < 0.05 at the 95% confidence interval (95% CI). The means of health expenditures were compared between independent groups using either Student’s t test or the Mann–Whitney U test. Analysis of variance and Kruskal–Wallis variance analysis methods were applied to compare the OOP health expenditures between independent variables. The distribution of OOP health expenditures was analyzed using the Kolmogorov–Smirnov test. A logistic regression model was used to determine the effect of independent variables on OOP health expenditures.

## Results

Two hundred thirty-two patients were evaluated in the study: 107 females (46.1%) and 125 males (53.9%). The consanguinity rate (139 patients, 59.9%) was high in our study. Consanguinity rate of parents living in rural areas of Ankara and Eastern part of Turkey were higher than parents living in the center of Ankara (*p* < 0.01). Forty-five (19.4%) patients had relatives with the same IEM. The mean age of the patients was 7.25 ± 4.82 years, with an age range of 0.3–18 years. Patients under 18 years of age following a diagnosis of IEM were included in the analysis. The type of IEM was amino acid metabolism disorders in 38.4% (n = 89), biotinidase deficiency in 14.7% (n = 34), lysosomal storage disorders in 12.5% (n = 29), carbohydrate metabolism disorders in 11.6% (n = 27), organic acidaemia in 9.9% (n = 23), urea cycle defects in 4.7% (n = 11), and rare types of inherited metabolic disorders in 8.2% (n = 19). Among them, phenylketonuria and biotinidase deficiency were detected earlier than other disorders because of newborn screening program in Turkey, *p* < 0.05. The mean age at diagnosis was 17.84 ± 33.76 months (0–180 months) in the study population. The majority of respondents (77.6%, n = 180) were insured by social security institutions, 16.4% (n = 38) had green cards, and 5.2% (n = 14) were uninsured (Table 1).

**Table 1:** Demographic characteristics of participants with ınborn errors of metabolism

N: 232		Mean ± standart deviation (SD) Median (min–max)	
Patients age (years)		7.25 ± 4.82 6.92(0.3–18)	
		N	%
*Gender*			
Girls		107	46.1
Boys		125	53.9
*Parental education levels*			
Unıversity		34	14.7
High school		88	37.9
Secondary school		58	25
Primary school		50	21.6
İlliterate		2	0.9
*Family type*			
Nuclear family		196	84.5
Extended family		36	15.5
Parental consanguinity		139	59.9
Patients' relatives had the same IEM		45	19.4
*Types of IEM*			
Amino acid metabolism disorders		89	38.4
Biotinidase deficiency		34	14.7
Lysosomal storage disorders		29	12.5
Carbohydrate metabolism disorders		27	11.6
Organic acidemia		23	9.9
Urea cycle defects		11	4.7
Rare types of inherited metabolic disorders		19	8.2
*Type of Insurence*			
Social Security Institution		180	77.6
Green card		38	16.4
Uninsured		14	5.2
*Metabolic unıt state*			
Center of Ankara		72	31
Rural part of Ankara		76	32.8
Outside of Ankara		84	36.2

The participants reported their education levels as 14.7% (n = 34) university, 37.9% (n = 88) high school, 25% (n = 58) middle school, 21.6% (n = 50) elementary school and 0.9% (n = 2) illiterate (Table 1).

A total of 196 caregivers (84.5%) stated that they were living in nuclear families, and 36 (15.5%) stated that they were living in extended families. The average monthly catastrophic costs per patient were notably higher in extended families (*p* < 0.05) (Table 2).

**Table 2:** Comparison of those who make catastrophic health expenditures and those who do not

	Catastrophic spending no		Catastrophic spending yes		Test statistic	p*
	N	%	N	%		
*Diagnosis*						
Amino acid metabolism disorders	76	85.4	13	14.6	χ^2^ = 14.861	0.005
*Family type*						
Nuclear	181	92.3	15	7.7	χ2 = 7.228	0.013
Extended	28	77.8	8	22.2		
*Living place*						
Ankara center	71	98.6	1	1.4	χ2 = 27.682	< 0.001
District of Ankara City center	73	96.1	3	3.9		
outside Ankara	43	81.1	10	18.9		
District outside Ankara	14	66.7	7	33.3		
Village outside Ankara	8	80	2	20		
Monthly** income						
≤ 4250 TL	97	86.6	15	13.4	χ2 = 7.041	0.056
4250–6400 TL	63	88.7	8	11.3		
6400–10,000 TL	33	100	0	0		
≥ 10,000 TL	16	100	0	0		
*Social security*						
SGK	168	93.3	12	6.7	χ2 = 15.520	0.003
Bağkur	10	83.3	2	16.7		
Green card	18	69.2	8	30.8		
None	13	92.9	1	7.1		
*Changing provinces for follow-up and treatment*						
Yes	65	77.4	19	22.6	χ2 = 23.800	< 0.001
No	144	97.3	4	2.7		

*Chi-square/ Fisher's Exact test, **$1 = 14,650 TL (1st Aprıl 2022 Central Bank rate of Republic of Türkiye)

*N: number of patients, *%: percentages of patients

The mean household income in the study population was 6000 (3500–20,000) Turkish Liras (TL). Considering household income, 48.3% had a monthly income below 4250 TL. The average monthly out-of-pocket payments were 1039.22 ± 1030.08 Turkish Liras (TL) per capita. Catastrophic healthcare expenditures were the greatest among the poorest households and decreased with an increase in income. When we consider the CHE exceeding 40% of household income, 9.9% (n = 23) of the parents of our study population made catastrophic health expenditures. The parents of patients with amino acid metabolism disorders had the maximum financial burden, and their CHEs were higher than those of patients with other IEMs (Table 2).

Of the total participants, 31% (n = 72) lived in the center of Ankara, where the hospital with a metabolic center is located, 32.8% (n = 76) lived in the rural part of Ankara, where the hospital with a metabolic unit is absent, and 36.2% (n = 84) lived outside Ankara and stated that they had difficulty finding accommodations and reported travel expenses. Catastrophic health expenditures were higher in participants who were living in the rural part of Ankara or living in other cities than in participants who were living in the center of Ankara (*p* < 0.01) (Table 2). Catastrophic health expenditures were lower in participants diagnosed by newborn screening program (PKU and BD) than in participants who were not found by Newborn Screening Program. Cases found by National Newborn Screening Program with PKU and BD are routinely consulted nearest pediatric metabolic centers. Catastrophic health expenditures are higher in those metabolic disorders not found by Newborn Screening Program because they are sent from different provinces of Turkey, except for the diseases screened.

Parents living in the center of Ankara had higher education levels with compared to the rural part of Ankara or living in other cities (*p* < 0.01).

## Discussion

To our knowledge, this is the first study investigating the medical and nonmedical financial burden of families with IEM in Turkey. A total of 232 patients and their families from different cities were interviewed to investigate the OOPCs for each family. Catastrophic Health Expenditure (CHE) is when OOPC was more than 10% of total consumption expenses or 40% of total non-consumption expenditure of the household [5, 6].

A study by Ravangard et al. evaluated household catastrophic health expenditure and its effective factors from Iran and showed that 16.48% of studied households had faced CHEs. Higher rates of CHEs were observed in households living in rented houses, households with disabled members and households with children under 5 years old [7]. In our study, we found that parents of patients with amino acid metabolism disorders with dietary treatment had significantly higher CHEs than those who had other types of IEM.

Catatrophic health expenditures may be reduced by increasing the use of supplementary health insurance coverage and increasing the support of the Social Security and State Welfare Organizations for households with IEM.

Centers specializing in IEM should also be increased in every area of Turkey. Specialized centers are especially condensed in Ankara and Western part of Turkey. Patients living in those parts of Turkey can easily reach health care services and this situation decreases the transport costs. Further studies in a large number of patients are needed to estimate the cost of IEM in Turkey, taking into account regional differences in OOPCs. There is a need to develop a methodology to perform transparent OOPC studies for IEM to inform policy decisions. Turkey’s health and social systems must improve systemic access to government-funded financial support and assistance services for people affected by IEM.

Turkey has a high rate of consanguineous marriage, which is nearly 24% [8]. In our study population, the consanguinity rate (139 patients, 59.9%) was quite high. Therefore, autosomal recessive diseases are expected to be seen more frequently, as in other chronic diseases, and the necessity of life-long treatment to maintain good metabolic control in patients with IEM brings many challenges. Inborn Errors of Metabolism that fall into this group also require regular follow-up to avoid worsening of the clinical condition. Living distant from the metabolic center makes it difficult to reach treatment. Absenteeism from the work of caregivers and expenses for transportation systems are major indirect OOPCs [9].

Telemedicine will be an appropriate and effective option to reduce out of pocket expenses for inborn errors of metabolism. Telemedicine can be simply defined as the use of information and communication technologies to provide persistence of health care services. It has been preferred by both health care providers and patients, especially for the follow-up of inherited metabolic diseases.Living in rural areas or long distances from metabolic centers. Telemedicine, including phone calls and online communication, could be recommended to ensure continued follow-up and reduced out-of-pocket expenses, especially indirect expenses, during the treatment of patients with IEM.

Yekedüz et al. reported the Pros and Cons of Telemedicine for Inherited Metabolic Disorders in Turkey during the COVID-19 pandemic and stated that more than half of the parents (67.6%) who lived in another city reported accommodation problems while coming to the hospital, and most believed telemedicine would save their time (93.1%) and money for travel (81.6%). Telemedicine is not routinely used in the health system in Turkey. Whole world interest in telemedicine during the COVID-19 pandemic [10–14].

In conclusion, the high rate of consanguineous marriages in Turkey, the development of screening programs, increased awareness of inherited metabolic diseases, and revolution in diagnostic methods face an increasing number of patients with IEM. We believe that our research provided useful and significant information about the financial burden on the family due to IEM. National newborn screening program appears to be conducted successfully in Turkey. In near future we hope to have a chance of expanded Newborn Screening by MS/MS and by this way the diseases will have greater chance to be determined. Health care financing strategies and policies targeted at the reduction in CHE due to IEM are urgently required in Turkey [15–17].

## Data Availability

Not applicable for that section.

## Abbreviations

NBS: Newborn screening
TL: Turkish Liras
IEM: Inborn errors of metabolism
OOPCs: Out-of-pocket costs
CHE: Catastrophic health expenditures
IQR: Interquartile range
OOP: Out of pocket
